# The association between baseline and long-term status of metabolic score for insulin resistance index and the incidence of cardiometabolic multimorbidity

**DOI:** 10.21203/rs.3.rs-5068082/v1

**Published:** 2025-07-30

**Authors:** Man Yang, Jia Liu, Suwen Shen, Qian Shen, Yaqi Liu, Yun Qian

**Affiliations:** The Affiliated Wuxi Center for Disease Control and Prevention of Nanjing Medical University, Wuxi Center for Disease Control and Prevention; The Affiliated Wuxi Center for Disease Control and Prevention of Nanjing Medical University, Wuxi Center for Disease Control and Prevention; Department of Medical Administration, Suzhou Industrial Park Medical and Health Management Center; The Affiliated Wuxi Center for Disease Control and Prevention of Nanjing Medical University, Wuxi Center for Disease Control and Prevention; The Affiliated Wuxi Center for Disease Control and Prevention of Nanjing Medical University, Wuxi Center for Disease Control and Prevention; The Affiliated Wuxi Center for Disease Control and Prevention of Nanjing Medical University, Wuxi Center for Disease Control and Prevention

**Keywords:** Cardiometabolic multimorbidity, METS-IR, insulin resistance, CHARLS, long-term status

## Abstract

**Background:**

Cardiometabolic multimorbidity (CMM) is concurrently associated with a reduction in life expectancy and an increased propensity for all-cause mortality. Our objective was to evaluate the correlations between the baseline and longitudinal metabolic score for insulin resistance index (METS-IR) and the incidence of cardiometabolic multimorbidity (CMM) within a middle-aged and older Chinese population cohort..

**Methods and Results:**

A total of 8,050 participants were enrolled and included in the analytical dataset for this study. Long-term status of METS-IR were defined as updated mean METS-IR and high METS-IR exposure duration. Updated mean METS-IR was defined as the mean of the two METS-IR measurements. High METS-IR exposure duration was defined as the times of visits with a high METS-IR among the 2 visits, quantified as 0 year, 2 years and 4 years according to the optimal cut points from the receiver operating characteristic curves of the two METS-IR measurements, respectively. The outcome was defined as the occurrence of CMM, characterized by the presence of two or more cardiometabolic disorders as self-reported by participants, encompassing conditions such as diabetes, stroke, and cardiac events. During 6-year visit, 540 participants experienced CMM. Substantially elevated incidences of CMM were observed in participants belonging to the highest tertiles of both baseline and updated mean METS-IR. After multivariable adjustment, the odds ratios (ORs) with 95% confidence intervals (CIs) for CMM were 2.94 (CI: 2.04–4.22) for those in the highest baseline METS-IR tertile and 3.26 (CI: 1.90–5.59) for those in the highest updated mean METS-IR tertile, relative to participants in the lowest tertiles. Multivariable-adjusted spline regression models showed a linear association of baseline METS-IR (*P*_linearity_ <0.0001) and updated mean METS-IR (*P*_linearity_ <0.0001) with CMM. Moreover, participants with 2 and 4 years high METS-IR exposure duration had increased risk of CMM (ORs [95% CIs]: 2.45 [1.52–3.96] and 3.46 [2.18–5.51], respectively), compared with the reference of those with unexposed group.

**Conclusion:**

This study proved elevated baseline METS-IR, updated mean METS-IR, especially high METS-IR exposure duration was associated with CMM incidence among middle-aged and older Chinese.

## Introduction

Multimorbidity, the most common chronic condition experienced by adults, which refers to the co-occurrence of multiple chronic disease or conditions^1^. It is becoming a global health challenge which have led to the decline in quality of life and greater use of health-care resources^2^. As one such representative multimorbidity, cardiometabolic multimorbidity (CMM) is defined as the co-existence of two or three cardiometabolic diseases (CMDs), which including diabetes mellitus (DM), stroke and heart disease^3^. Previous studies from around the world has consistently shown that CMM is linked to a shortened lifespan and an increased risk of all-cause mortality^3–5^. Therefore, the efforts to prevent CMM by reducing risk factors are of substantial relevance to both public health initiatives and medical practice.

Insulin resistance (IR) signifies a decline or dysfunction in insulin sensitivity within peripheral tissues, as evidenced by compromised glucose uptake and oxidative metabolism. In addition, as a metabolic risk factor, IR itself is a prominent characteristic of cardiovascular and metabolic diseases, including hyperglycemia, atherosclerosis, diabetes mellitus and sroke^6–8^. While the euglycaemic-hyperinsulinaemic clamp (EHC) is broadly acknowledged as the gold-standard method and benchmark for evaluating IR, its adoption in routine clinical practice may be difficult due to its inherent complexities, time-intensive nature, and substantial resource requirements^9^. Thus, it is particularly important to recognize rapidly available and reliable IR markers. Recently, a number of methodologies incorporating simple routine biochemical markers have been introduced for the assessment of IR, including indices such as the Triglyceride Glucose (TyG) index and the ratio of triglycerides to high-density lipoprotein cholesterol (TG/HDL)^10,11^. Nevertheless, these indices overlook the influence of cholesterol levels and nutritional status on the pathogenesis of the disease.

Recently, the metabolic score for insulin resistance (METS-IR) index has been developed, which combined fasting plasma glucose (FPG), fasting triglycerides (TG), fasting high density lipoprotein cholesterol (HDL-C) and body mass index (BMI) mirroring nutritional status. This index has been shown to exhibit a high degree of agreement with the EHC^12^. Moreover, accumulative researches suggested that METS-IR was related to cardiometabolic disorders^13–15^. However, these preceding investigations have exclusively examined the association between METS-IR and specific diseases, furthermore, the assessment of METS-IR was conducted at a single time point, limiting the scope of the findings. Therefore, we aimed to explore the association of baseline and long-term MET-IR with CMM using data from the China Health and Retirement Longitudinal Study (CHARLS).

## Methods

### Study participants

The research involved a sample of middle-aged and elderly individuals from the CHARLS—a persistent, nationally representative, prospective, and longitudinal investigation based on the population of China. Details of the study design have been published elsewhere^16^. A total of 17,708 participants from 10,257 households across 28 provinces in China were enrolled at the baseline phase (2011–2012, wave 1), utilizing a multistage stratified probability proportional-to-size sampling approach. Participants in the CHARLS were monitored biennially through in-person interviews facilitated by computer-assisted personal interviewing technology. Four consecutive follow-ups were executed from 2013–2014 (wave 2), 2015–2016 (wave 3), and 2017–2018 (wave 4) for the enduring participants. Ethical approval for all the CHARLS waves was granted from the Institutional Review Board (IRB) at Peking University. The IRB approval number for the main household survey, including anthropometrics, is IRB00001052–11015; the IRB approval number for biomarker collection, was IRB00001052–11014. The details of the CHARLS data are available on the website (http://charls.pku.edu.cn/en).

**Figure 1:**
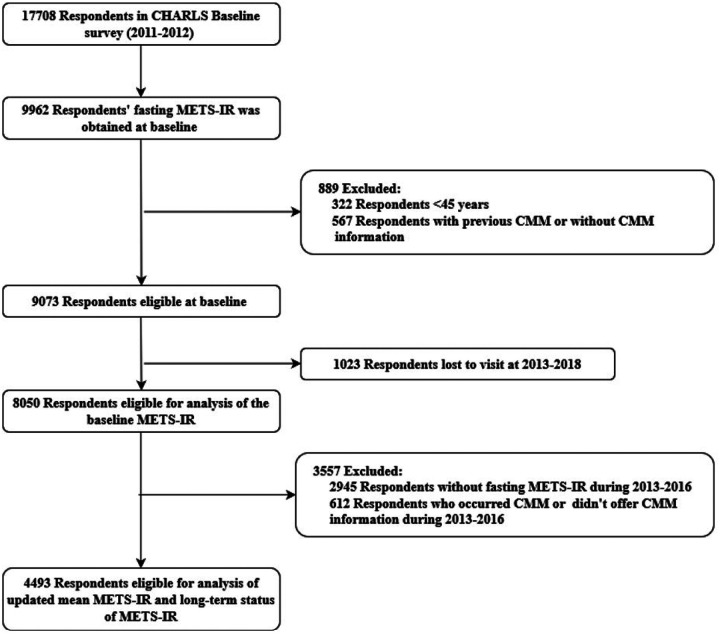
Flow chart of participants’ selection.

In our study, participants were excluded if they had missing survey information on the MET-IR at baseline (i.e., 2011–2012) or if they had known physician-diagnosed CMM (e.g., diabetes mellitus, stroke or cardiac events) during 2011–2015. We first included 9962 participants with fasting METS-IR information at baseline and then excluded 322 participants <45 years, 567 participants with previous CMM or without CMM information and 1023 participants lost to follow up. A total of 8050 respondents were eligible for analysis of the baseline METS-IR. In subsequent analysis of updated mean METS-IR and high METS-IR exposure duration, we further excluded 2945 participants without fasting METS-IR information and 612 participants who occurred CMM or didn’t offer CMM information during 2013–2016. Finally, a total of 4493 participants were eligible for the analysis of updated mean METS-IR and high METS-IR exposure duration (Fig. 1). The CHARLS was granted ethical clearance by the Institutional Review Board of Peking University. All participants provided written informed consent prior to their inclusion in the study. The research adhered to the Strengthening the Reporting of Observational Studies in Epidemiology (STROBE) guidelines for transparent and comprehensive reporting.

### Data collection and measurements

Baseline data encompassing demographic characteristics, lifestyle risk factors, medical history, and medication utilization were gathered from the participants. History of chronic disease was determined based on participants’ self-disclosures of diagnoses confirmed by healthcare professionals. Three blood pressure (BP) was measured with an electronic sphygmomanometer (Omron HEM-7200 Monitor) after 5 min of rest in the sitting position. Mean of the three BP measurements was used in the analyses. High inflammatory status was defined as high-sensitivity C-reactive protein (hsCRP) ≥ 3 mg/L according to previous studies^17^. Assessment of renal function was based on estimated glomerular filtration rate (eGFR) calculated using the Chronic Kidney Disease Epidemiology Collaboration creatinine equation with adjusted coefficient of 1.1 for the Chinese population^18^. In accordance with the Kidney Disease: Improving Global Outcomes guidelines^19^, we defined normal renal function as eGFR ≥ 90 ml/min/1.73 m^2^ and abnormal renal function as eGFR ≥ 90 ml/min/1.73 m^2^. Grip strength from the dominant hand was determined using a handgrip dynamometer (YuejianTM WL-1000 dynamometer) twice, and the average was used. Participants were instructed to perform five consecutive sit-to-stand repetitions on a chair at their quickest pace, with the chair-rising time being meticulously recorded using a stopwatch for each repetition^20^. The summary score for the balance test was categorized as follows: a score of 1 was assigned if the participant failed to complete either the semi-tandem or side-by-side tests to the maximum duration; a score of 2 was given for those who did not complete the semi-tandem test to the maximum time but managed to complete the side-by-side test to the maximum duration; a score of 3 was designated for participants who completed the semi-tandem test to the maximum duration but were unable to finish the full-tandem test within the expected time; and a score of 4 was allocated to those who completed both the semi-tandem and full-tandem tests to the maximum duration^21^.

Blood samples were meticulously collected from each participant by proficient medical personnel from the Chinese Center for Disease Control and Prevention (China CDC), adhering to a standardized protocol. Participants were instructed to undergo an overnight fast prior to blood collection; however, blood samples were also obtained from those who had not fasted, with their fasting status meticulously recorded as a variable within the dataset. Over 92% of respondents who gave blood reported that they were fasting. Complete blood count (CBC) test was measured on automated analyzers available at county CDC stations or town/village health centers. All the plasma samples including triglyceride (TG), high-density lipoprotein-cholesterol (HDL-C), fasting glucose (FBG) were measured at the Youanmen Center for Clinical Laboratory of Capital Medical University. Body mass index (BMI) was caculated as weight divided by height squared (kg/m^2^).

METS-IR was calculated as (ln(2*FBG +TG)*BMI))/(ln(HDL-C)^12^. The MET-IR measurements obtained during two visits (wave 1 and wave 3) from CHARLS were utilized to evaluate the long-term status of the METS-IR. Long-term status of METS-IR were defined as updated mean METS-IR and high METS-IR exposure duration. In the present analysis, updated mean METS-IR was defined as the mean of the METS-IR measurements from 2 visits. High METS-IR exposure duration was defined as the times of visits with a high METS-IR (over the cutoff mentioned in the Statistical analysis) among the 2 visits, quantified as 0 year, 2 years and 4 years.

### Assessment of CMM

The identification of chronic conditions diagnosed by physicians was determined through self-reported data collected at the baseline assessment and subsequent follow-up surveys (for diabetes or high blood sugar, “Have you been diagnosed with diabetes or high blood sugar by a doctor?”, for heart attack, coronary heart disease, or other heart problems, “Have you been diagnosed with heart attack, coronary heart disease, angina, congestive heart failure, or other heart problems by a doctor?” and for stroke, “Have you been diagnosed with stroke by a doctor?”). Participants who responded affirmatively to the pertinent query were deemed to have a cardiometabolic condition. Of those, CMM was defined as participants who reported suffering from at least two cardiometabolic conditions^22^.

### Statistical analysis

Participants in the study were categorized into tertiles based on their baseline METS-IR values, the updated mean METS-IR values, as well as across three distinct exposure durations characterized by high METS-IR, respectively. Baseline demographic and clinical attributes were examined across the various participant groups. The optimal cutoff for METS-IR associated with CMM incidence was determined using the receiver operating characteristic curves of METS-IR at wave 1 (≥34.81) and wave 3 (≥36.87), respectively.

Multivariable logistic regression models were used to estimate the risk of CMM associated with baseline METS-IR, updated mean METS-IR and high METS-IR exposure duration, respectively. Tests for linear trend in risk across baseline METS-IR, updated mean METS-IR and high METS-IR exposure duration were performed using these category as continuous variables. The odds ratios (ORs) and 95% confidence intervals (CIs) were computed for each 1-standard deviation (SD) increase in both baseline and updated mean METS-IR. All analyses were conducted with adjustments for the following covariates: age, sex, rural residency, current smoking status, and current alcohol consumption in Model 1; adding medical history (hypertension, dyslipidemia, chronic kidney disease, malignant tumor, lung disease, liver disease, stomach/digestive disease, arthritis, asthma, psychological problem, memory problem) and medicine history (taking any medicine or treatment for hypertensive, dyslipidemia and malignant tumor) in model 2; adding waist circumference (WC), systolic BP, low-density lipoproteins cholesterol (LDL-C), eGFR, uric acid (UC) and hsCRP in model 3; adding dominant hand grip strength, chair-rising time, lung function peak flow and balance test summary score in model 4. The impact of both baseline and updated mean METS-IR on the risk for cardiometabolic multimorbidity (CMM) was investigated utilizing ordinal logistic regression analyses. Nonparametric restricted cubic splines were used to examine the shape of the association of baseline METS-IR and updated mean METS-IR with CMM with 3 knots (at the 10th, 50th and 90th percentiles of baseline METS-IR and updated mean METS-IR, respectively). We assessed the capacity of baseline METS-IR, updated mean METS-IR, and the duration of high METS-IR exposure to enhance risk stratification when added to a basic model that includes well-established risk factors. The Net Reclassification Improvement (NRI) and the Integrated Discrimination Improvement (IDI) were computed to quantify this effect^23^.

To test the robustness of our findings, we performed several sensitivity analyses by excluding (1) participants with death, (2) participants with abnormal BMI (≥ 28 kg/m2), FBG (≥ 126 mg/dL), TG (≥150mg/dL) and HDL-C (< 35 mg/dL). Furthermore, we conducted subgroup analyses, stratified by sex, age (<65 and ≥65 years), current smoking, current drinking, history of hypertension and dyslipidemia, baseline hsCRP (<3.0 mg/L and ≥3.0 mg/L) and baseline eGFR (<90 ml/min/1.73 m^2^ and ≥ 90 ml/min/1.73 m^2^) in multivariable adjusted logistic regression models. This study did not employ sample weights, as prior research has demonstrated comparable outcomes whether weights were applied or not^24,25^. Two-sided *P* value <0.05 was considered to be significant. Data analysis was performed using SAS statistical software (version 9.4).

## Results

### Baseline characteristics

A total of 8050 participants (4330 men and 3720 women; mean age 58.78±9.08 years) were enrolled in the current study. Participants with high baseline METS-IR, in contrast to those with low METS-IR, were more frequently younger, female, and residing in urban areas. They also exhibited lower rates of smoking and alcohol consumption, as well as a reduced use of anti-hypertensive and lipid-lowering medications. Moreover, this group had a higher incidence of hypertension and dyslipidemia, but a lower occurrence of lung disease, stomach or digestive disorders, and asthma. They also demonstrated higher values for BMI, waist circumference (WC), systolic blood pressure (SBP), blood glucose, low-density lipoprotein cholesterol (LDL-C), uric acid (UC), high-sensitivity C-reactive protein (hsCRP), and grip strength of the dominant hand (Table 1). Similar results were observed when participants were categorized by updated mean METS-IR and high METS-IR exposure duration (Table 2 and 3).)

### Association of baseline METS-IR with incident CMM

**Figure 2:**
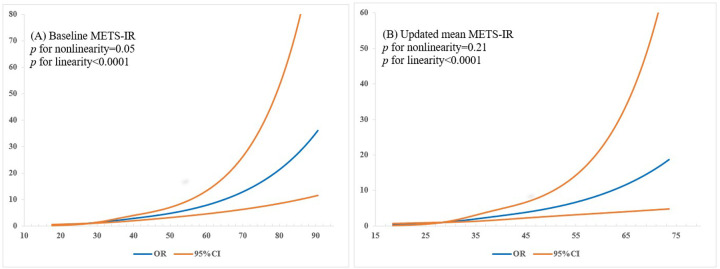
Adjusted odds radios of CMM according to METS-IR in respondents. (A) baseline METS-IR; (B) updated mean METS-IR. Odds ratios and 95% confidence intervals derived from restricted cubic spline regression, with knots placed at the 10th, 50th and 90th percentiles of METS-IR. The reference point for METS-IR is the median of the first tertile (baseline METS-IR: 27.92; updated mean METS-IR: 28.25). Odds radios were adjusted for the same variables as model 4 in Table 4 unless the variable was excluded.

Over the course of a 6-year follow-up period, 540 participants (6.71%) developed CMM. Notably, the incidence of CMM was significantly greater in the subset of participants with higher baseline METS-IR values. After adjustment for potential confounding factors, with each SD (9.22) of METS-IR increasing, the risk of incident CMM elevated by 82% (OR: 1.82, 95%CI: 1.57–2.11). In the categorical analysis, the multivariable-adjusted ORs for CMM in the T2 and T3 groups, as compared to the T1 group, were 1.54 (95% CI: 1.10–2.17) and 2.94 (95% CI: 2.04–4.22), respectively, with a statistically significant trend (P_trend_ < 0.0001) (Table 4). Analysis using multivariable-adjusted spline regression models revealed a linear relationship between baseline METS-IR and the incidence of CMM (*P*_linearity_ <0.0001) (Fig. 2A). Adding baseline METS-IR tertiles to a model containing conventional risk factors significantly improved risk reclassification for CMM (continuous NRI was 29.11% [p <0.0001] and IDI was 0.53% [p =0.002]) (Table 5).

### Association of long-term status of METS-IR with incident CMM

Similarly, a significant association was found between the updated mean METS-IR and incident CMM when the aforementioned analysis was replicated. The multivariable-adjusted ORs and 95% CIs for the highest tertile of updated mean METS-IR relative to the lowest tertile were 3.17 (95% CI: 1.49–6.75) for the incidence of CMM. Each 1-SD (9.62) increment in updated mean METS-IR was associated with 99% (OR: 1.99, 95%CI: 1.53–2.59) increased risk of CMM (Table 4). Multivariable-adjusted spline regression models showed a linear association of updated mean METS-IR with CMM incidence (*P*_linearity_ <0.0001) (Fig. 2B). Incorporating updated mean METS-IR tertiles into a model that already includes conventional risk factors yielded a significant enhancement in the risk reclassification for CMM (continuous NRI was 40.18% [p <0.0001] and IDI was 0.62% [p =0.01]) (Table 5).

The incidence rates of cardiometabolic multimorbidity (CMM) were elevated to 6.23% and 8.91% at 2-year and 4-year durations of high METS-IR exposure, respectively, compared to the unexposed group (0-year exposure) which had an incidence rate of 2.63%. After multivariable adjustment, compared with unexposed group (0 year), risk of CMM was significantly higher in those with 2 years group (OR: 2.45, 95%CI: 1.52–3.96) and 4 years group (OR: 3.46, 95%CI: 2.18–5.51), respectively (Table 4). Adding three high METS-IR exposure duration to a model containing conventional risk factors significantly improved risk reclassification for CMM (continuous NRI was 40.56% [*p* <0.0001] and IDI was 0.76% [*p* = 0.006]) (Table 5).

### Additional analyses

Sensitivity analyses yielded results aligned with the primary analysis. Even after excluding participants who had deceased by Wave 4 and limiting the sample to those with normal levels of BMI, FBG, TG, and HDL-C, the findings remained consistent with the initial analysis (Table 6).

In order to delve deeper into the association between the baseline and long-term status of METS-IR and the incidence of CMM, a series of subgroup analyses were performed. None of the subgroups, including the sex, age, current drinking status, current smoking status, history of hypertension, history of dyslipidemia, high-sensitivity C-reactive protein and estimated glomerular filtration rate subgroups, profoundly changed the relationship between the baseline and long-term status of METS-IR and CMM incidence (all P for interaction>0.05) (Table 7–9).

## Discussion

Our prospective study insights into the prognostic significance of baseline and prolonged METS-IR status in predicting cardiometabolic risk among middle-aged and elderly individuals of the Chinese population. The findings not only contribute to the extension of knowledge regarding the longevity of cardiometabolic risk but also enhance our understanding of the underlying pathophysiological mechanisms. In this prospective study among CHARLS participants, we documented that elevated baseline METS-IR, updated mean METS-IR and high METS-IR exposure duration during follow-up was independently associated with increased risk of CMM. The integration of baseline or long-term METS-IR measurements into the conventional risk factors model significantly enhanced the risk reclassification for CMM, as indicated by the NRI and IDI. Moreover, the observed association remained robust following both sensitivity analyses and examination within various subgroups. To the best of our knowledge, this is the first study to prospectively evaluate the predictive significance of both baseline and long-term status of METS-IR on the incidence of cardiometabolic diseases among middle-aged and older adults in China, utilizing data from a nationally representative panel.

METS-IR, a new non-insulin-based index, is calculated on clinical markers including FBG, TG, HDL-C and BMI. METS-IR is a practical alternative to insulin-related indices, which is primarily due to the fact that serum insulin levels are not regularly assessed in routine clinical settings. Previous studies were usually based on predicting role of METS-IR in the development of specific disease, including CVD and DM. In a study consisted of 6489 participants aged 35–70 years without a history of CVD during a median follow-up of 10.6 years, elevated METS-IR was found to be independently associated with incident CVD^14^. A study conducted in Mexico City showed that individuals who developed type 2 diabetes mellitus (T2DM) had elevated baseline METS-IR, and the likelihood of developing incident T2DM increased consistently with higher percentile METS-IR^12^. Aforementioned researches were based on a single METS-IR measurement, only a few studies are based on the change or long-term status of METS-IR. Tian et al. found that the potential for future CVD incidence was linked to the cumulative exposure to METS-IR, as well as the timing and progression of METS-IR^26^. Similarly, data from a rural Chinese area showed that an elevation in METS-IR and a decrease in METS-IR over a 6-year period were both independently associated with an increased risk of developing T2DM^27^. Nevertheless, the existing research has largely focused on evaluating the relationship between METS-IR and specific health outcomes within select a certain group of population, rather than examining the broader implications of METS-IR for cardiometabolic health among the general community-dwelling population.

It is worth mentioning that the influence of baseline and long-term status of METS-IR on CMM has not been ascertained. Taking into account the variability of METS-IR over time, which might engender regression dilution bias and and thereby impact the accuracy of the findings, employing serial assessments of long-term METS-IR is likely to yield results that are both more reliable and more robust, enhancing the overall validity of the finding. In the present study, the dose-response association still existed when using updated mean METS-IR and long-term status of METS-IR to evaluate. More importantly, the updated mean METS-IR and long-term status of METS-IR seemed to be more significantly associated with CMM than baseline METS-IR. Consequently, our findings underscore the importance of long-term surveillance of METS-IR within clinical settings, suggesting that such monitoring could be instrumental in extending the period of METS-IR remission among middle-aged and elderly individuals of the Chinese population.

Although the precise mechanisms underlying the association between METS-IR and CMM are not yet fully elucidated, several speculative explanations have been suggested. One explanation suggests that considering the involvement of BMI, METS-IR might be a superior indicator for assessing IR in adipose tissue, muscle, and the liver^28^. Abundant adipose tissue not only elevates metabolic risk but is also linked to elevated blood glucose concentrations and reduced levels HDL-C^29^. Hypertriglyceridemia exacerbates this by raising free fatty acid (FFA) levels, which can disrupt insulin signaling and prompt oxidative stress in the tissues, leading to IR in the bone and live^30^. Inflammations caused by high cumulative IR could affect blood glucose and enhance the formation of atherosclerosis-associated foam cells and vulnerable plaques^31–33^. Another explanation suggests that the increased platelet aggregation, adhesion, and activation, as indicated by the higher IR, contributed to the occlusion of blood vessels, leading to disturbances in hemodynamic^34,35^.

Our research boasts several notable strengths, including the extensive scale and national representativeness of the prospective study conducted across China, coupled with an impressive participant response rate and rigorous adjustment for potential confounders within our multivariable models. As a result, the present investigation affords high-caliber evidence regarding the association between METS-IR and cardiometabolic risk. Several limitations also need to be mentioned. First, The CHARLS study was conducted exclusively on a Chinese population, and thus, the findings drawn from our research may not be directly applicable or generalizable to diverse populations. Second, CMM was derived from participants’ self-reported physician diagnoses, which may cause information bias, although this method has been widely adopted in epidemiologic study^30^. Third, the METS-IR was not measured in wave 2 or wave 4, precluding the analysis of its trajectory over time. Moreover, it is important to note that the current analysis was not preplanned as part of the original study protocol. This observational analysis could be influenced by potential biases and confounding factors which we did not account for in our adjustments. Consequently, the outcomes of our investigation primarily serve to postulate hypotheses that will need to be explored and validated in subsequent research initiatives.

In conclusion, this study proved elevated baseline METS-IR, especially high METS-IR exposure duration was associated with CMM incidence among middle-aged and older Chinese. Our findings indicate that this simple index may be useful for identifying individuals at high risk CMM in advance, and emphasize the importance in long-term monitoring of METS-IR in clinical practice.

## Figures and Tables

**Table 1.** Characteristics of the study participants according to the tertiles of baseline METS-IR

Characteristics	Baseline METS-IR			*p* value
	<30.96	<30.96	<30.96	
Subjects, n (%)	2656 (32.99)	2655 (32.98)	2739 (34.03)	
**Demographics**				
Age, years	60.88 ± 9.56	58.22 ± 8.84	57.28 ± 8.44	<0.0001
Male, n (%)	1387 (52.22)	1224 (46.10)	1109 (40.49)	<0.0001
Rural, n (%)	1978 (74.47)	1783 (67.16)	1537 (56.12)	<0.0001
Current smoking, n (%)	996 (38.06)	779 (29.71)	616 (22.70)	<0.0001
Current drinking, n (%)	1027 (38.68)	911 (34.33)	758 (27.69)	<0.0001
**Medical history**				
Hypertension, n (%)	418 (15.80)	578 (21.87)	1030 (37.70)	<0.0001
Dyslipidemia, n (%)	102 (3.89)	179 (6.84)	410 (15.26)	<0.0001
Chronic kidney disease, n (%)	153 (5.80)	155 (5.86)	134 (4.90)	0.23
Malignant tumor, n (%)	18 (0.68)	20 (0.75)	27 (0.99)	0.42
Lung disease, n (%)	338 (12.75)	213 (8.04)	202 (7.38)	<0.0001
Liver disease, n (%)	96 (3.63)	87 (3.30)	98 (3.59)	0.77
Stomach/digestive disease, n (%)	666 (25.13)	610 (22.98)	516 (18.85)	<0.0001
Arthritis, n (%)	906 (34.16)	892 (33.62)	985 (36.04)	0.15
Asthma, n (%)	142 (5.36)	97 (3.67)	99 (3.63)	0.002
Psychological problem, n (%)	34 (1.28)	37 (1.40)	29 (1.06)	0.53
Memory problem, n (%)	30 (1.13)	33 (1.24)	33 (1.21)	0.93
**Medicine history**				
Anti-hypertensive drugs, n (%)	260 (9.83)	406 (15.37)	806 (29.50)	<0.0001
Lipid-lowering drugs, n (%)	40 (1.53)	78 (2.98)	227 (8.45)	<0.0001
Oncology drugs or treatment, n (%)	15 (0.56)	13 (0.49)	23 (0.84)	0.23
**Clinical features**				
Body mass index, kg/m^2^	20.08 (18.87, 21.23)	23.21 (22.07, 24.42)	26.76 (25.08, 28.67)	<0.0001
Waist circumference, cm	76.40 (72.20, 81.00)	84.20 (80.00, 89.00)	93.45 (88.40, 99.00)	<0.0001
Systolic blood pressure, mmHg	121.50 (110.00, 136.50)	124.50 (112.50, 139.00)	130.50 (118.00, 145.00)	<0.0001
Blood glucose, mg/dL	98.28 (91.62, 106.20)	101.16 (94.32, 109.62)	106.02 (98.10, 117.90)	<0.0001
Low-density lipoproteins cholesterol, mg/dL	111.34 (91.24, 113.93)	117.53 (97.42, 139.56)	117.14 (94.72, 142.27)	<0.0001
Estimated glomerular filtration rate, ml/min/1.73 m^2^	106.54 (96.98, 113.93)	107.99 (98.32, 115.65)	107.65 (96.65, 114.87)	0.0006
Uric acid, mg/dL	4.11 (3.46, 4.94)	4.21 (3.54, 5.03)	4.53 (3.78, 5.40)	<0.0001
High-sensitivity C-reactive protein, mg/L	0.74 (0.43, 1.55)	0.92 (0.52, 1.89)	1.35 (0.75, 2.69)	<0.0001
Dominant hand grip strength, kg	30.00 (24.00, 37.00)	31.00 (25.00, 40.00)	31.20 (25.00, 40.00)	<0.0001
Chair-rising time, s	10.18 (8.07, 12.60)	10.05 (7.97, 12.54)	10.03 (8.19, 12.50)	0.54
Lung function peak flow, ml	260.00 (180.00, 360.00)	300.00 (210.00, 380.00)	300.00 (210.00, 380.00)	<0.0001
Balance test summary score	4.00 (4.00, 4.00)	4.00 (4.00, 4.00)	4.00 (4.00, 4.00)	0.71

Continuous variables are expressed as mean ± SD (normal distribution) or median (interquartile range) (not normal distribution) and compared using F tests (normal distribution) or Wilcoxon rank-sum tests (not normal distribution) as appropriate.

Categorical variables are expressed as number (percent) and compared using χ^2^ tests.

**Table 2.** Characteristics of the study participants according to the tertiles of updated mean METS-IR.

Characteristics	Updated mean METS-IR			*p* value
	<31.18	31.18–37.31	≥37.31	
Subjects, n (%)	1484 (33.03)	1481 (32.96)	1528 (34.01)	
**Demographics**				
Age, years	60.85 ± 8.88	58.10 ± 8.49	56.95 ± 8.05	0.0003
Male, n (%)	820 (55.26)	652 (44.02)	610 (39.92)	<0.0001
Rural, n (%)	1131 (76.21)	994 (67.21)	893 (58.44)	<0.0001
Current smoking, n (%)	584 (39.92)	414 (28.24)	337 (22.27)	<0.0001
Current drinking, n (%)	891 (39.85)	500 (33.760	427 (27.96)	<0.0001
**Medical history**				
Hypertension, n (%)	232 (15.72)	310 (21.02)	563 (36.94)	<0.0001
Dyslipidemia, n (%)	56 (3.83)	112 (7.70)	216 (14.30)	<0.0001
Chronic kidney disease, n (%)	74 (5.02)	80 (5.43)	74 (4.86)	0.77
Malignant tumor, n (%)	7 (0.47)	11 (0.74)	11 (0.72)	0.59
Lung disease, n (%)	196 (13.23)	124 (8.38)	114 (7.47)	<0.0001
Liver disease, n (%)	55 (3.73)	44 (2.98)	47 (3.09)	0.47
Stomach/digestive disease, n (%)	363 (24.51)	337 (22.77)	276 (18.09)	<0.0001
Arthritis, n (%)	485 (32.73)	480 (32.43)	528 (34.60)	0.39
Asthma, n (%)	80 (5.41)	55 (3.72)	58 (3.81)	0.04
Psychological problem, n (%)	21 (1.42)	16 (1.09)	16 (1.05)	0.59
Memory problem, n (%)	22 (1.49)	5 (0.34)	10 (0.66)	0.002
**Medicine history**				
Anti-hypertensive drugs, n (%)	150 (10.16)	198 (13.42)	426 (27.95)	<0.0001
Lipid-lowering drugs, n (%)	22 (1.50)	52 (3.57)	124 (8.21)	<0.0001
Oncology drugs or treatment, n (%)	9 (0.61)	7 (0.47)	12 (0.79)	0.55
**Clinical features**				
Body mass index, kg/m^2^	20.04 (18.88, 21.18)	23.15 (22.04, 24.32)	26.71 (25.11, 28.74)	<0.0001
Waist circumference, cm	76.40 (72.00, 81.00)	84.00 (80.00, 88.60)	93.60 (88.40, 99.00)	<0.0001
Systolic blood pressure, mmHg	121.00 (109.50, 135.00)	123.50 (112.00, 138.50)	130.00 (117.50, 145.00)	<0.0001
Blood glucose, mg/dL	99.00 (91.80, 107.10)	100.26 (94.14, 108.54)	104.40 (96.84, 113.58)	<0.0001
Low-density lipoproteins cholesterol, mg/dL	110.18 (89.69, 131.06)	116.37 (96.65, 139.56)	117.53 (95.10, 140.72)	<0.0001
Estimated glomerular filtration rate, ml/min/1.73 m^2^	107.36 (98.48, 113.96)	108.70 (99.40, 115.94)	108.29 (97.69, 115.20)	0.008
Uric acid, mg/dL	4.08 (3.43, 4.92)	4.11 (3.51, 4.96)	4.43 (3.67, 5.3)	<0.0001
High-sensitivity C-reactive protein, mg/L	0.72 (0.44, 1.57)	0.89 (0.52, 1.83)	1.27 (0.70, 2.39)	<0.0001
Dominant hand grip strength, kg	30.00 (24.30, 37.00)	31.00 (25.00, 39.60)	32.00 (25.50, 40.00)	<0.0001
Chair-rising time, s	10.12 (8.00, 12.60)	10.12 (8.01, 12.62)	10.12 (8.29, 12.62)	0.52
Lung function peak flow, ml	260.00 (190.00, 360.00)	300.00 (210.00, 380.00)	300.00 (220.00, 380.00)	<0.0001
Balance test summary score	4.00 (4.00, 4.00)	4.00 (4.00, 4.00)	4.00 (4.00, 4.00)	0.31

Continuous variables are expressed as mean ± SD (normal distribution) or median (interquartile range) (not normal distribution) and compared using F tests (normal distribution) or Wilcoxon rank-sum tests (not normal distribution) as appropriate.

Categorical variables are expressed as number (percent) and compared using χ^2^ tests.

**Table 3.** Characteristics of the study participants according to the high MET-IR exposure duration.

Characteristics	High MET-IR exposure duration			*p* value
	0 year	2 years	4 years	
Subjects, n (%)	2243 (49.92)	802 (17.85)	1448 (32.23)	
**Demographics**				
Age, years	59.94 ± 8.90	58.44 ± 8.38	56.67 ± 7.95	<0.0001
Male, n (%)	1158 (51.63)	349 (43.52)	575 (39.71)	<0.0001
Rural, n (%)	1653 (73.70)	519 (64.71)	846 (58.43)	<0.0001
Current smoking, n (%)	801 (36.20)	207 (26.07)	327 (22.79)	<0.0001
Current drinking, n (%)	844 (37.64)	268 (33.42)	406 (28.06)	<0.0001
**Medical history**				
Hypertension, n (%)	360 (16.13)	215 (26.88)	530 (36.73)	<0.0001
Dyslipidemia, n (%)	104 (4.70)	76 (9.67)	204 (14.26)	<0.0001
Chronic kidney disease, n (%)	115 (5.16)	46 (5.76)	67 (4.64)	0.50
Malignant tumor, n (%)	10 (0.45)	8 (1.00)	11 (0.76)	0.20
Lung disease, n (%)	267 (11.92)	59 (7.37)	108 (7.46)	<0.0001
Liver disease, n (%)	78 (3.49)	23 (2.88)	45 (3.12)	0.65
Stomach/digestive disease, n (%)	548 (24.46)	176 (21.97)	252 (17.43)	<0.0001
Arthritis, n (%)	732 (32.66)	264 (32.96)	497 (34.37)	0.55
Asthma, n (%)	106 (4.74)	33 (4.13)	54 (3.74)	0.33
Psychological problem, n (%)	31 (1.39)	9 (1.12)	13 (0.90)	0.40
Memory problem, n (%)	27 (1.21)	1 (0.13)	9 (0.62)	0.009
**Medicine history**				
Anti-hypertensive drugs, n (%)	231 (10.35)	141 (17.63)	402 (27.86)	<0.0001
Lipid-lowering drugs, n (%)	45 (2.03)	35 (4.45)	118 (8.25)	<0.0001
Oncology drugs or treatment, n (%)	10 (0.45)	6 (0.75)	12 (0.83)	0.31
**Clinical features**				
Body mass index, kg/m^2^	20.97 (19.46, 88.33)	23.88 (22.69, 25.17)	26.76 (25.16, 28.79)	<0.0001
Waist circumference, cm	78.80 (74.00, 83.20)	86.55 (82.00, 91.00)	93.80 (88.20, 99.00)	<0.0001
Systolic blood pressure, mmHg	122.00 (110.00, 135.50)	125.50 (114.00, 140.00)	130.00 (117.00, 144.50)	<0.0001
Blood glucose, mg/dL	99.18 (92.16, 107.10)	102.06 (95.04, 110.16)	104.22 (96.57, 113.49)	<0.0001
Low-density lipoproteins cholesterol, mg/dL	112.89 (92.78, 133.76)	116.37 (93.56, 139.67)	117.14 (95.49, 141.50)	0.0002
Estimated glomerular filtration rate, ml/min/1.73 m^2^	107.55 (98.67, 114.53)	108.15 (97.38, 115.20)	108.71 (98.47, 115.78)	0.10
Uric acid, mg/dL	4.08 (3.42, 4.93)	4.22 (3.62, 5.03)	4.40 (3.68, 5.30)	<0.0001
High-sensitivity C-reactive protein, mg/L	0.77 (0.45, 1.64)	0.97 (0.56, 1.99)	1.26 (0.70, 2.38)	<0.0001
Dominant hand grip strength, kg	30.00 (24.50, 38.00)	30.50 (25.00, 39.00)	32.00 (25.50, 40.00)	<0.0001
Chair-rising time, s	10.07 (7.99, 12.60)	10.24 (8.03, 12.72)	10.11 (8.34, 12.57)	0.48
Lung function peak flow, ml	270.00 (200.00, 365.00)	295.00 (200.00, 370.00)	300.00 (220.00, 390.00)	<0.0001
Balance test summary score	4.00 (4.00, 4.00)	4.00 (4.00, 4.00)	4.00 (4.00, 4.00)	0.15

Continuous variables are expressed as mean ± SD (normal distribution) or median (interquartile range) (not normal distribution) and compared using F tests (normal distribution) or Wilcoxon rank-sum tests (not normal distribution) as appropriate.

Categorical variables are expressed as number (percent) and compared using χ^2^ tests.

**Table 4.** Odds ratios and 95% confidence intervals of CMM according to METS-IR.

	Case (%)	Age, sex-adjusted	Model 1	Model 2	Model 3	Model 4
**Baseline METS-IR**						
T1 (<30.96)	75 (2.82)	1.00	1.00	1.00	1.00	1.00
T2 (30.96–37.56)	139 (5.24)	2.04 (1.53–2.72)	2.04 (1.52–2.72)	1.92 (1.42–2.60)	1.71 (1.23–2.36)	1.54 (1.10–2.17)
T3 (≥37.56)	326 (11.90)	5.10 (3.92–6.23)	5.03 (3.85–6.57)	3.68 (2.76–4.92)	3.19 (2.26–4.50)	2.94 (2.04–4.22)
*p* for trend		<0.0001	<0.0001	<0.0001	<0.0001	<0.0001
Each SD (9.22) increase		1.77 (1.62–1.94)	1.75 (1.59–1.92)	1.42 (1.28–1.58)	1.82 (1.59–2.09)	1.82 (1.57–2.11)
**Updated mean METS-IR**						
T1 (<31.18)	38 (2.56)	1.00	1.00	1.00	1.00	1.00
T2 (31.18–37.31)	62 (4.19)	1.76 (1.16–2.67)	1.80 (1.19–2.75)	1.73 (1.12–2.67)	1.52 (0.95–2.42)	1.56 (0.93–2.61)
T3 (37.31)	138 (9.03)	4.12 (2.83–6.00)	4.33 (2.95–6.36)	3.50 (2.32–5.28)	2.95 (1.79–4.86)	3.26 (1.90–5.59)
*p* for trend		<0.0001	<0.0001	<0.0001	<0.0001	<0.0001
Each SD (9.62) increase		1.38 (1.17–1.64)	1.39 (1.17–1.65)	1.19 (1.07–1.32)	1.95 (1.52–2.50)	1.99 (1.53–2.59)
**High METS-IR exposure duration**						
0 year	59 (2.63)	1.00	1.00	1.00	1.00	1.00
2 years	50 (6.23)	2.54 (1.73–3.75)	2.67 (1.80–3.95)	2.46 (1.64–3.70)	2.39 (1.53–3.72)	2.45 (1.52–3.96)
4 years	129 (8.91)	3.91 (2.83–5.40)	4.11 (2.95–5.73)	3.37 (2.36–4.81)	3.07 (1.99–4.76)	3.46 (2.18–5.51)
*p* for trend		<0.0001	<0.0001	<0.0001	<0.0001	<0.0001

Optimal cut points for METS-IR at wave 1 (≥34.81) and wave 3 (≥36.87) were obtained from the receiver operating characteristic curves.

Model 1: adjusted for age, sex, rural region, current smoking, current drinking.

Model 2: adjusted for model 1 + medical history (hypertension, dyslipidemia, diabetes mellitus, chronic kidney disease, malignant tumor, lung disease, liver disease, stomach/digestive disease, arthritis, asthma, psychological problem, memory problem) + medicine history (taking any medicine or treatment for hypertensive, dyslipidemia, diabetes mellitus, cardio-cerebrovascular disease and malignant tumor).

Model 3: adjusted for model 2 + body mass index, waist circumference, systolic blood pressure, low-density lipoproteins cholesterol, blood glucose, estimated glomerular filtration rate, uric acid and high-sensitivity C-reactive protein.

Model 4: adjusted for model 3 + dominant hand grip strength, chair-rising time, lung function peak flow and balance test summary score.

**Table 5.** Reclassification statistics for CMM by METS-IR.

	Continuous NRI (95% CI), %	*P* value	IDI (95% CI), %	*P* value
Conventional model	Reference		Reference	
Conventional model + baseline METS-IR tertiles	29.11 (19.08–39.14)	<0.0001	0.53 (0.19–0.87)	0.002
Conventional model + updated mean METS-IR tertiles	40.18 (25.71–54.65)	<0.0001	0.62 (0.14–1.10)	0.01
Conventional model + high MET-IR exposure duration	40.56 (25.95–55.18)	<0.0001	0.76 (0.22–1.29)	0.006

Abbreviations: CI = confidence interval; NRI = net reclassification improvement; IDI = integrated discrimination index.

Optimal cut points for METS-IR at wave 1 (≥34.81) and wave 3 (≥36.87) were obtained from the receiver operating characteristic curves.

Conventional model included age, sex, rural region, current smoking, current drinking, active physical activity, medical history (hypertension, dyslipidemia, chronic kidney disease, malignant tumor, lung disease, liver disease and stomach/digestive disease), medicine history (taking any medicine or treatment for hypertensive, dyslipidemia and malignant tumor), systolic blood pressure, low-density lipoproteins cholesterol, estimated glomerular filtration rate, high-sensitivity C-reactive protein, dominant hand grip strength, chair-rising time, lung function peak flow and balance test summary score unless the variable was excluded.

**Table 6.** Odds ratios and 95% confidence intervals of CMM to METS-IR in sensitivity analysis.

	Case (%)	Age, sex-adjusted	Model 1	Model 2	Model 3	Model 4
**Baseline METS-IR**						
T1 (<30.96)	63 (2.84)	1.00	1.00	1.00	1.00	1.00
T2 (30.96–37.56)	95 (5.12)	2.06 (1.48–2.86)	2.03 (1.45–2.84)	1.78 (1.25–2.54)	1.89 (1.27–2.82)	1.76 (1.15–2.68)
T3 (≥37.56)	46 (8.08)	3.45 (2.31–5.14)	3.37 (2.24–5.07)	2.32 (1.48–3.61)	2.59 (1.54–4.37)	2.53 (1.44–4.42)
*p* for trend		<0.0001	<0.0001	<0.0001	<0.0001	<0.0001
Each SD (9.22) increase		2.61 (1.99–2.43)	2.58 (1.96–3.41)	1.95 (1.45–2.63)	2.16 (1.51–3.08)	2.18 (1.49–3.19)
**Updated mean METS-IR**						
T1 (<31.18)	33 (2.71)	1.00	1.00	1.00	1.00	1.00
T2 (31.18–37.31)	43 (4.06)	1.67 (1.04–2.67)	1.65 (1.02–2.67)	1.51 (0.91–2.52)	1.77 (1.01–3.14)	1.87 (0.99–3.53)
T3 (37.31)	28 (6.81)	2.99 (1.76–5.08)	3.04 (1.77–5.22)	2.14 (1.18–3.89)	2.74 (1.38–5.46)	3.17 (1.49–6.75)
*p* for trend		<0.0001	<0.0001	<0.0001	<0.0001	<0.0001
Each SD (9.62) increase		2.48 (1.67–3.69)	2.52 (1.68–3.80)	1.83 (1.19–2.83)	2.13 (1.28–3.55)	2.44 (1.38–4.35)
**High MET-IR exposure duration**						
0 year	50 (2.72)	1.00	1.00	1.00	1.00	1.00
2 years	29 (6.76)	2.72 (1.89–4.38)	2.79 (1.72–4.52)	2.46 (1.46–4.14)	2.78 (1.57–4.93)	2.80 (1.49–5.26)
4 years	25 (5.95)	2.58 (1.56–4.27)	2.67 (1.60–4.46)	1.98 (1.14–.344)	2.46 (1.32–4.58)	2.93 (1.50–5.71)
*p* for trend		<0.0001	<0.0001	<0.0001	<0.0001	<0.0001

Optimal cut points for METS-IR at wave 1 (≥34.81) and wave 3 (≥36.87) were obtained from the receiver operating characteristic curves.

Model 1: adjusted for age, sex, rural region, current smoking, current drinking.

Model 2: adjusted for model 1 + medical history (hypertension, dyslipidemia, diabetes mellitus, chronic kidney disease, malignant tumor, lung disease, liver disease, stomach/digestive disease, arthritis, asthma, psychological problem, memory problem) + medicine history (taking any medicine or treatment for hypertensive, dyslipidemia, diabetes mellitus, cardio-cerebrovascular disease and malignant tumor).

Model 3: adjusted for model 2 + body mass index, waist circumference, systolic blood pressure, low-density lipoproteins cholesterol, blood glucose, estimated glomerular filtration rate, uric acid and high-sensitivity C-reactive protein.

Model 4: adjusted for model 3 + dominant hand grip strength, chair-rising time, lung function peak flow and balance test summary score.

**Table 7.** Subgroup analysis of ORs (95 % CIs) of CMM according to baseline METS-IR.

METS-IR	T1	T2	T3	*p*_interaction_
	<30.96	30.96–37.56	≥37.56	
**Sex**				0.45
Male	1.00	1.54 (0.93–2.55)	3.32 (1.92–5.76)	
Female	1.00	1.62 (1.01–2.60)	2.81 (1.72–4.60)	
**Age**				0.89
<65	1.00	1.89 (1.21–2.95)	3.18 (1.98–5.09)	
≥65	1.00	1.18 (0.66–2.11)	3.10 (1.67–5.75)	
**Current drinking**				0.35
Yes	1.00	1.58 (0.87–2.89)	3.37 (1.78–6.37)	
No	1.00	1.55 (1.02–2.36)	2.85 (1.83–4.45)	
**Current smoking**				0.08
Yes	1.00	2.00 (1.06–3.77)	4.94 (2.44–10.01)	
No	1.00	1.38 (0.91–2.08)	2.44 (1.60–3.74)	
**History of hypertension**				
Yes	1.00	1.32 (0.79–2.19)	2.08 (1.23–3.52)	0.02
No	1.00	1.62 (1.01–2.61)	3.61 (2.16–6.04)	
**History of dyslipidemia**				0.35
Yes	1.00	1.46 (0.52–4.12)	1.83 (0.64–5.22)	
No	1.00	1.52 (1.05–2.20)	3.17 (2.15–4.68)	
**High-sensitivity C-reactive protein**				0.73
<3.0 mg/L	1.00	1.53 (1.05–2.23)	2.72 (1.81–4.07)	
≥3.0 mg/L	1.00	1.52 (0.63–3.67)	3.66 (1.49–9.03)	
**Estimated glomerular filtration rate**				0.86
<90 ml/min/1.73 m^2^	1.00	1.78 (0.75–4.19)	2.88 (1.19–7.01)	
≥90 ml/min/1.73 m^2^	1.00	1.50 (1.03–2.19)	2.92 (1.95–4.37)	

NA: the sample is not enough.

In the multivariate models, confounding factors such age, sex, rural region, current smoking, current drinking, active physical activity, medical history (hypertension, dyslipidemia, chronic kidney disease, malignant tumor, lung disease, liver disease and stomach/digestive disease), medicine history (taking any medicine or treatment for hypertensive, dyslipidemia and malignant tumor), systolic blood pressure, low-density lipoproteins cholesterol, estimated glomerular filtration rate, high-sensitivity C-reactive protein, dominant hand grip strength, chair-rising time, lung function peak flow and balance test summary score were included unless the variable was used as a subgroup variable.

**Table 8.** Subgroup analysis of ORs (95 % CIs) of CMM according to updated mean METS-IR.

Updated mean METS-IR	T1	T2	T3	*p*_interaction_
	<31.18	31.18–37.31	≥37.31	
**Sex**				0.22
Male	1.00	1.74 (0.79–3.84)	5.37 (2.37–12.14)	
Female	1.00	1.44 (0.72–2.91)	2.20 (1.05–4.62)	
**Age**				0.21
<65	1.00	1.88 (0.96–3.71)	3.92 (1.96–7.81)	
≥65	1.00	1.63 (0.68–3.94)	2.67 (0.96–7.43)	
**Current drinking**				0.75
Yes	1.00	1.55 (0.67–3.58)	3.25 (1.33–7.95)	
No	1.00	1.60 (0.82–3.12)	3.47 (1.74–6.94)	
**Current smoking**				0.25
Yes	1.00	1.38 (0.51–.73)	6.78 (2.42–18.96)	
No	1.00	1.63 (0.87–3.06)	2.54 (1.32–4.91)	
**History of hypertension**				0.13
Yes	1.00	1.26 (0.56–2.84)	2.28 (1.02–5.10)	
No	1.00	1.73 (0.86–3.48)	3.90 (1.81–8.44)	
**History of dyslipidemia**				0.99
Yes	1.00	1.13 (0.19–6.73)	2.32 (0.42–12.71)	
No	1.00	1.67 (0.96–2.90)	3.56 (1.99–6.35)	
**High-sensitivity C-reactive protein**				0.32
<3.0 mg/L	1.00	1.79 (1.00–3.22)	3.59 (1.93–6.68)	
≥3.0 mg/L	1.00	0.89 (0.28–2.85)	1.84 (0.55–6.13)	
**Estimated glomerular filtration rate**				0.59
<90 ml/min/1.73 m^2^	1.00	1.42 (0.37–5.54)	1.71 (0.37–7.85)	
≥90 ml/min/1.73 m^2^	1.00	1.64 (0.93–2.89)	3.65 (2.03–6.56)	

NA: the sample is not enough.

In the multivariate models, confounding factors such age, sex, rural region, current smoking, current drinking, active physical activity, medical history (hypertension, dyslipidemia, chronic kidney disease, malignant tumor, lung disease, liver disease and stomach/digestive disease), medicine history (taking any medicine or treatment for hypertensive, dyslipidemia and malignant tumor), systolic blood pressure, low-density lipoproteins cholesterol, estimated glomerular filtration rate, high-sensitivity C-reactive protein, dominant hand grip strength, chair-rising time, lung function peak flow and balance test summary score were included unless the variable was used as a subgroup variable.

**Table 9.** Subgroup analysis of ORs (95 % CIs) of CMM according to high MET-IR exposure duration.

High MET-IR exposure duration	0 year	2 years	4 years	*p*_interaction_
**Sex**				0.09
Male	1.00	2.86 (1.30–6.26)	6.21 (3.04–12.70)	
Female	1.00	2.17 (1.16–4.06)	2.19 (1.17–4.09)	
**Age**				0.30
<65	1.00	3.34 (1.83–6.08)	4.08 (2.29–7.26)	
≥65	1.00	1.82 (0.71–4.68)	3.00 (1.17–7.74)	
**Current drinking**				0.96
Yes	1.00	2.23 (0.95–5.25)	3.85 (1.72–8.60)	
No	1.00	2.65 (1.46–4.80)	3.53 (1.98–6.31)	
**Current smoking**				0.13
Yes	1.00	2.74 (1.01–7.47)	8.00 (3.25–19.69)	
No	1.00	2.38 (1.35–4.17)	2.60 (1.49–4.54)	
**History of hypertension**				0.08
Yes	1.00	2.12 (1.02–4.39)	2.50 (1.24–5.03)	
No	1.00	2.40 (1.23–4.69)	4.18 (2.19–7.98)	
**History of dyslipidemia**				0.92
Yes	1.00	3.03 (0.64–14.29)	3.12 (0.75–13.02)	
No	1.00	2.39 (1.42–4.03)	3.61 (2.19–5.96)	
**High-sensitivity C-reactive protein**				0.37
<3.0 mg/L	1.00	2.54 (1.48–4.35)	3.50 (2.06–5.95)	
≥3.0 mg/L	1.00	2.22 (0.72–6.86)	2.58 (0.91–7.34)	
**Estimated glomerular filtration rate**				0.36
<90 ml/min/1.73 m^2^	1.00	1.93 (0.52–7.10)	1.53 (0.38–6.07)	
≥90 ml/min/1.73 m^2^	1.00	2.63 (1.55–4.45)	3.96 (2.39–6.56)	

In the multivariate models, confounding factors such age, sex, rural region, current smoking, current drinking, active physical activity, medical history (hypertension, dyslipidemia, chronic kidney disease, malignant tumor, lung disease, liver disease and stomach/digestive disease), medicine history (taking any medicine or treatment for hypertensive, dyslipidemia and malignant tumor), systolic blood pressure, low-density lipoproteins cholesterol, estimated glomerular filtration rate, high-sensitivity C-reactive protein, dominant hand grip strength, chair-rising time, lung function peak flow and balance test summary score were included unless the variable was used as a subgroup variable.

## Data Availability

This analysis uses data or information from the Harmonized CHARLS dataset and Codebook, Version D as of June 2021 developed by the Gateway to Global Aging Data. The development of the Harmonized CHARLS was funded by the National Institute on Ageing (R01AG030153, RC2AG036619, R03 AG043052). For more information, please refer to www.g2aging.org.
